# Pediatric Posttraumatic Endophthalmitis in China for Twenty Years

**DOI:** 10.1155/2017/5248767

**Published:** 2017-01-16

**Authors:** Yan Sheng, Wen Sun, Yangshun Gu, Andrzej Grzybowski

**Affiliations:** ^1^Department of Ophthalmology, The First Affiliated Hospital, School of Medicine, Zhejiang University, Hangzhou, Zhejiang, China; ^2^Department of Ophthalmology, Poznan City Hospital, Poznan, Poland; ^3^Department of Ophthalmology, University of Warmia and Mazury, Olsztyn, Poland

## Abstract

Pediatric posttraumatic endophthalmitis (PTE) is a rare but serious disease that frequently has a poor visual prognosis. To date, only a few English studies have focused on this disease. We perform a systematic review of the Chinese literature on pediatric PTE and describe the epidemiology, management, causative organisms, and visual acuity outcomes of reported cases in twenty years. We found that* Staphylococcus epidermidis* was the most common isolated organism and the use of a disposable syringe needle was the most common cause of ocular injuries in pediatric PTE in China. In the last ten years, the time from injury to first presentation for treatment has shortened, the proportion of cases resulting from a disposable syringe needle has decreased, the use of intravitreal antibiotics as the initial treatment has increased, and the use of palliative treatment has decreased. Although these favorable changes have occurred in the last ten years, the visual prognosis of pediatric PTE is still poor.

## 1. Introduction

Pediatric endophthalmitis is a rare but serious disease that frequently has a poor visual prognosis. The most common cause is ocular trauma [1–3]. To date, only a few English studies have focused on pediatric endophthalmitis after trauma [1–9], possibly because of its low incidence [1–3]. Other than one recent large sample from India [8], all other reported samples were small.

In China, the incidence of pediatric endophthalmitis after open globe injury ranges from 9.7% to 20.6% [10–14], which is higher than that in developed countries (2.5% [1] and 3.5% [3]). At the same time, China is the world's most populous country. As a result, more pediatric patients have been diagnosed with posttraumatic endophthalmitis (PTE) in China and articles related to such patients have been published in Chinese journals. However, only one study involving a small sample of Chinese pediatric PTE has recently been published in an English journal [9].

The purpose of the present study was to perform a systematic review of the Chinese literature on pediatric PTE and to describe the epidemiology, management, causative organisms, and visual acuity (VA) outcomes of reported cases. A second aim was to examine changes in etiology, treatment, and visual outcomes in patients with pediatric PTE in China over time.

## 2. Methods

A systematic literature search was conducted in April 2016 of the China National Knowledge Infrastructure (CNKI), Wanfang, Weipu, and PubMed databases, using the following search terms: “pediatric or child or children or adolescent or infant,” “trauma or injury,” “endophthalmitis,” and “China.” The search was limited to references published within the last 20 years in the English or Chinese languages. A total of 79 articles were retrieved. After a detailed examination of these articles, we included only those that focused on a clinical study of pediatric PTE with a follow-up of more than 3 months. Articles were excluded if the content included other types of pediatric endophthalmitis or the article was a review or nursing study. After these selections, 25 articles were retained for further systematic analysis. We collected published data that included the name of the first author, publication year, number of patients and eyes, age and gender of patients, etiology, type of ocular trauma, time from injury to first presentation, initial VA, therapeutic methods, VA outcome, and causative organisms. The information provided in each article is shown in Table 1 [9, 15–38]. Because there were no articles meeting the criteria for randomized controlled trials and the study design, inclusion criteria, and therapeutic methods in these articles varied widely, it was impossible to conduct a meta-analysis. Instead, we collected the published data from each article in a database (Excel) and calculated the median or proportions for age, gender, type of ocular trauma, treatment methods, causative organisms, and VA outcomes. In addition, we divided the 25 articles into two periods according to the year in which the case data were collected (1994–2003 and 2004–2013) and compared the characteristics of the cases between these two periods.

Statistical analyses were performed with SPSS software (SPSS 15.0). Pearson chi-square tests were used to determine the differences in the proportions of treatment methods, causative agents, initial VA, and final VA between 1994–2003 and 2004–2013. An independent sample *t*-test was used to compare the time from injury to presentation for treatment between the two periods.* P* values of <0.05 were considered statistically significant.

## 3. Results

The 25 [9, 15–38] selected articles included 820 children with PTE. All cases were unilateral. The proportion of male to female children was 2.2 : 1 (567 male to 253 female). The children ranged in age from 1 to 14 years. The mean age as reported in 21 articles was 7.0 years for 670 children.

### 3.1. Time from Injury to First Presentation for Treatment

Seventeen articles provided information on the time between injury and first presentation in 599 cases, the mean being 9.3 days (range 4 hours to 120 days).

### 3.2. Nature of Trauma

All cases of PTE resulted from an open globe injury. Twenty-one articles provided information on the mechanism of injury in 704 eyes. In accordance with the standardized classification of ocular trauma by Kuhn et al. [42], in 619 of these eyes (87.9%) the cause was laceration, including penetrating, perforating, and intraocular foreign body wounds, and in 85 eyes (12.1%) the cause was rupture. All 25 articles reported the cause of injury. The leading cause was a disposable syringe needle (43.8%, 250/571). Other common causes included metal objects (scissors, wire, and nail), wood (tree branch, bamboo stick, and pencil), and an explosion (14.3%, 82/571). The injury site was reported in 10 articles for 220 eyes. Of these eyes, 179 (81.4%) had corneal wounds, 17 (7.7%) had scleral wounds, and 24 (10.9%) had corneoscleral wounds.

At the time of diagnosis with endophthalmitis, of 655 eyes, 421 (64.3%) had a cataract, as reported in 19 articles, and of 595 eyes, 122 (20.5%) had a retinal detachment, as reported in 17 articles.

### 3.3. Presenting VA

Twenty-one articles reported the presenting VA of 577 PTE cases. It was <5/200 in 78.0% (450/577) of cases, with no light perception in 9.9% (57/577), light perception in 25.5% (147/577), hand movements in 25.6% (148/577), and counting fingers in 17.0% (98/577). A total of 111 cases (19.2%) had a presenting VA of ≥5/200. The residual 16 patients (2.8%) were too young to obtain a VA.

### 3.4. Causative Organisms

Of the 14 articles with information on cultures from intraocular samples, 8 reported performing bacterial and fungal cultures for 297 eyes, and 6 reported performing a bacterial culture alone for 197 eyes. Positive bacterial results were obtained in 36.2% of eyes. Of the 160 eyes with an identified bacterial isolate, 126 (78.8%) had Gram-positive bacteria and 34 (21.2%) had Gram-negative bacteria. More details about bacterial cultures are shown in Table 2.

### 3.5. Management and Treatment

Of the 25 articles, 15 (507 eyes) reported pars plana vitrectomy (PPV) as the single method of PTE treatment. In the other 10 articles (313 eyes), the reported treatment methods for PTE included intravitreal antibiotics, PPV, and palliative treatment (intravenous and topical antibiotics). In these 10 articles, 192 cases (61.3%) were initially treated with PPV. Intravitreal antibiotics were administered as the initial treatment in 82 cases (26.2%), and 69 of the 82 cases underwent further vitrectomy. In the remaining 39 cases (12.5%), only palliative treatment was given.

In 15 articles, a second PPV operation was performed in 44 of 449 cases (9.8%). Of these 44 cases, 40 (90.9%) were retinal detachment after the first PPV, 3 (6.8%) had uncontrolled inflammation that did not respond to the first PPV, and 1 (2.3%) was a serious vitreous hemorrhage after the first PPV.

### 3.6. VA Outcomes

The final VA was reported in 23 articles regarding 678 cases. After we excluded 14 cases in which the VA could not be tested, there remained 664 cases with a final recorded VA. Compared with initial vision, the final VA improved in 497 cases (74.9%), was unchanged in 123 cases (18.5%), and had decreased in 44 cases (6.6%). Of 664 eyes, 206 (31.0%) achieved a VA of 20/200 or better, which included 48 eyes that were better than 20/40; 333 eyes (50.1%) had a VA of counting fingers or worse, which included 78 eyes (11.7%) with no light perception vision.

In 20 articles, 45 of 607 patients developed phthisis bulbi at the last follow-up. Of these patients, 9 had been managed without intraocular injection and vitrectomy and 13 had developed postoperative retinal detachment but reoperation was refused. Another 16 of the 519 patients experienced persistent hypotony after vitrectomy.

### 3.7. Trend Changes between the Two Periods

We divided the 25 articles into two periods (1994–2003 and 2004–2013) according to the year in which the case data were collected and compared characteristics of the cases between these two periods.

The mean time from injury to first presentation for treatment shortened significantly from 13.4 days to 6.4 days (*P* < 0.05) between the two periods (Table 3). Use of a disposable syringe needle was responsible for 57.7% of cases in the first ten years (1994–2003), compared with 22.0% in the second ten years (2004–2013) (*P* < 0.05) (Table 3). Metal objects, such as scissors, wires, and nails, were the leading cause of injury in the second ten years (2004–2013).

Excluding articles in which all cases of PTE were treated with PPV, 10 articles reported other treatment methods. In these cases, the use of intravitreal antibiotics as the initial treatment increased from 12.0% in 1994–2003 to 38.0% in 2004–2013 (*P* < 0.05), and the use of palliative treatment decreased significantly, from 27.5% to 0% (*P* < 0.05) (Table 3). The proportion of PPV use as the initial treatment between the two periods was similar (60.5% to 61.9%, *P* > 0.05).

For the initial VA, there was no significant difference between the two periods (*P* > 0.05). The final VA was improved in the second ten years compared with the first ten years (*P* < 0.05), and the proportion of phthisis bulbi decreased from 10.4% in 1994–2003 to 7.8% in 2004–2013 (*P* < 0.05) (Table 3).

## 4. Discussion

In this study, the mean time from injury to presentation for treatment was 9.7 days. In most of these cases, no primary repair had been performed before presentation with PTE. Al-Rashaed and Abu El-Asrar [4] reported that the primary repair had been performed in 62.8% of eyes at the time of diagnosis of PTE. Another study [5] reported that the primary repair of ocular injury in 7 of 10 cases of pediatric PTE was performed less than 3 days following trauma and the mean time was 4 days. Compared with these reports, the mean time of primary repair in the present study was even longer. The most likely reason was that a disposable syringe needle was the main cause of injury (43.8%) of ocular trauma, which usually leads to a painless and self-sealing wound. In addition, because they are afraid of being scolded or they lack language expression, children typically do not inform the family of the trauma in time. Thus, most children with a penetrating needle injury in this study were not seen for medical care until inflammatory symptoms appeared, such as pain, redness, or vision loss. These factors can result in a delay in diagnosis and treatment.

Two characteristics were commonly related to the nature of trauma in the present study. One is that a disposable syringe needle was the most common cause of ocular injuries, being responsible for approximately half of the cases. In developed countries, PTE following a penetrating needle injury has rarely been reported. Two studies from the United States that reported the causative agents of PTE in children showed that only 1 of 19 cases was due to needle injury [2, 5]. A penetrating needle injury seems to be more common in developing countries. A report from Turkey found that 8.3% of 242 perforating ocular injuries in children were caused by injection needles [43]. Several studies from India also reported ocular injuries in children following penetration with hypodermic needles [39, 40]. In one of these reports, the author pointed out that endophthalmitis occurred more frequently in syringe needle cases, most having been used during medical care; the microorganisms that subsist on these needles were the most important reason for the development of endophthalmitis [39]. In the present study, most needles were medical waste that had been disposed of inadequately. Thus, inadequate disposal of syringe needles is an important ocular hazard for children in China.

The other common characteristic of injuries in the present study was that 14.3% of them resulted from an explosion that resulted in rupture or an intraocular foreign body. To our knowledge, previous reports on PTE in children have not reported cases following an explosion. The pediatric explosive injuries in our study were always caused by setting off firecrackers, which is a traditional Chinese custom that children enjoy and participate in. Therefore, explosion is a common cause of ocular injury in China. In two large-sample studies from China, 140 of 836 (16.7%) ocular injuries in children were due to explosion [44, 45]. At the same time, ocular explosion injuries were often associated with IOFB, which is a risk factor for PTE [41]. Severe ocular rupture with delayed primary repair may even result in PTE.

The culture results in the present study showed that Gram-positive organisms (77.2%) constituted the majority of pathogens. This result is consistent with previous reports of pediatric PTE in which the proportion of Gram-positive organisms ranged from 66.6% to 100% [1–5]. In the present study,* Staphylococcus epidermidis* was isolated most commonly, accounting for 31.2% of the 160 isolates. In the pooled data from developed countries [1–5],* Streptococcus* species were found to be the most common organism, accounting for 57.1% of cases (32/56), whereas* Staphylococcus epidermidis* accounted for only 14.3% (Table 4). Because* Staphylococcus epidermidis* is part of the skin's normal flora, an open wound permits its entry into the eye. Delayed primary repair may increase the risk of a* Staphylococcus epidermidis* infection. In the present study, the mean time from injury to first presentation in these cases was 9.7 days, which was significantly longer than in other studies [1–5]. This finding may explain the higher proportion of* Staphylococcus epidermidis* in the present study. In a recent study from India that reported 143 PTE cases in children,* Enterococcus faecalis* was the most common causative organism. The difference in common causative organisms in different countries may be related to the various characteristics of nature and of the environment in different countries.

PTE in children generally has a poor prognosis. Pooled data from seven previous studies of pediatric PTE shows that only 35 of 94 cases (37.5%) achieved a VA better than 20/200 (Table 5) [2–5, 39–41]. In the present study, only 31.7% of the cases (184/581) had VAs of 20/200 or better, which was similar to that of previous studies. Factors that influence the prognosis in adult PTE have been analyzed extensively; they include being affected by the virulence of the microbe-causing infection, the presence or absence of a retinal break or detachment, the time of treatment, the presence or absence of an IOFB, and the extent of the initial injury [46]. Recently, one large-sample study from India reported that corneal abscess and retinal detachment are associated with a poor outcome. As the current study is a review of the literature, we cannot identify which factors influenced the prognosis in pediatric PTE.

By comparing the data between two periods, we found that the proportion of disposable syringe needle injury decreased from 57.7% to 22.0% and the time from injury to first presentation shortened significantly from 13.4 days to 6.4 days. These changes may suggest that, in the past ten years, the inadequate disposal of syringe needles has gradually improved and the protection of children in society has increased in China. At the same time, the use of intravitreal antibiotics as the initial treatment increased from 12.0% to 38.0% and palliative treatment decreased from 27.5% to 0%. This wide variation in treatment protocols during the study period reflects an improvement in medical facilities and treatment strategies in China. During the first ten-year period (1994–2003), PPV was not performed in most primary hospitals and general treatment guidelines for pediatric PTE were lacking in China. In the second ten-year period (2004–2013), prompt intraocular injection and timely PPV surgery had been widely adopted for the treatment of PTE among Chinese doctors, promoting more reasonable and standardized management of the disease. Nonetheless, expert consensus or unified guidelines for the treatment of pediatric PTE in China remain to be developed.

*Staphylococcus epidermidis* was the most common isolated organism and the use of a disposable syringe needle was the most common cause of ocular injuries in pediatric PTE in China. Although some favorable changes in treatment methods, causes of injury, presentation for treatment, and final outcome of VA have occurred in the last ten years, the visual prognosis of pediatric PTE is still poor. This study is limited by the fact that it is a literature review. Well-designed, randomized, controlled studies are required to determine the most effective treatments and their prognostic differences for pediatric PTE.

## Figures and Tables

**Table 1:** Data available for analysis in the 25 articles.

First author	Year of publication	Number of eyes	Age	Gender	Time from injury to visiting	Etiology	Type of ocular trauma	Site of injury	Initial visual acuity (VA)	Treatment method	Culture result	Final VA	Follow-up time
Dong et al. [15]	1998	33	+	+	+	+			+	+	+	+	*+*
Wang [16]	1999	19	+	+	+	+			+	+		+	*+*
Fang [17]	2000	18	+	+	+	+	+	+	+	+		+	*+*
Gong et al. [18]	2000	28	+	+	+	+	+		+	+		+	*+*
Zhu and Zhang [19]	2003	68	+	+	+	+	+		+	+		+	*+*
Meng et al. [20]	2003	18	+	+	+	+	+			+	+	+	*+*
Li and Song. [21]	2004	20	+	+	+	+	+			+		+	*+*
Chen et al. [22]	2005	17	+	+		+	+	+	+	+	+	+	*+*
Liu et al. [23]	2005	24	+	+	+	+	+	+	+	+		+	*+*
Wang et al. [24]	2005	50	+	+	+	+	+	+	+	+	+	+	*+*
Zhou and Qiao [25]	2005	13	+	+		+	+		+	+		+	*+*
Sun et al. [26]	2006	12	+	+	+	+	+	+	+	+	+	+	*+*
Sha [27]	2006	25	+	+		+		+	+	+	+	+	*+*
Zhou et al. [28]	2007	103	+	+		+	+			+	+		*+*
Meng et al. [29]	2007	26	+	+	+	+	+		+	+	+	+	*+*
Shi [30]	2007	23	+	+	+	+	+		+	+	+	+	*+*
Peng et al. [31]	2008	16	+	+		+	+	+	+	+		+	*+*
Ge [32]	2008	78	+	+	+	+	+		+	+		+	*+*
Jiang et al. [33]	2010	39	+	+	+	+			+	+	+		*+*
Wu and Xiao [34]	2011	102	+	+	+	+	+			+	+	+	*+*
Wei [35]	2012	17	+	+	+	+	+		+	+	+	+	*+*
Shuang-Hua et al. [36]	2012	13	+	+		+	+		+	+		+	*+*
Lei et al. [37]	2013	19	+	+		+	+	+	+	+		+	*+*
Gong and Jiang [38]	2015	24	+	+	+	+	+	+	+	+	+	+	*+*
Wu et al. [9]	2016	15	+	+		+	+	+	+	+	+	+	*+*

Total		820	25	25	17	25	21	10	21	25	14	23	25

**Table 2:** Results of bacterial cultures.

Bacteria	Eyes (*n*)	Percent (%)
Gram-positive coccus	99	61.9
* Staphylococcus epidermidis*	50	31.2
* Staphylococcus aureus *	36	22.5
* Streptococcus *spp.	10	6.3
* Pneumococcus*	3	1.9
Gram-positive bacillus	27	16.9
* Bacillus *spp.	13	8.1
* Propionibacterium*	4	2.5
* Bacillus diphtheriae*	5	3.1
* Corynebacterium*	3	1.9
* Nocardia*	1	0.6
* Listeria*	1	0.6
Gram-negative bacillus	30	18.7
* Escherichia coli*	10	6.2
* Pseudomonas aeruginosa*	6	3.8
* Klebsiella *spp.	6	3.8
* Proteus mirabilis*	3	1.9
* Bacillus levans*	3	1.9
* Haemophilus*	1	0.6
* Acinetobacter *spp.	1	0.6
Gram-negative coccus	4	2.5
* Neisseria*	4	2.5

Total	160	100

**Table 3:** Comparison of characteristics of posttraumatic endophthalmitis cases between 1994–2003 and 2004–2013.

	1994–2003	2004–2013	*P*
Time from injury to presentation			
Number of articles reporting	8 (260 cases)	6 (220 cases)	
Mean time	13.4 days	6.4 days	<0.001
Causative agents			
Number of articles	9 (234 cases)	7 (236 cases)	
Disposable syringe needle	135 (57.7%)	52 (22%)	<0.001
Initial visual acuity			0.369
Number of articles reporting	9 (268 cases)	9 (169 cases)	
NLP and LP	113 (42.2%)	62 (36.7%)	
HM and CF	116 (43.3%)	75 (44.4%)	
≥5/200	39 (14.5%)	32 (18.9%)	
Final visual acuity			0.042
Number of articles reporting	11 (306 cases)	8 (218 cases)	
**≥**20/200	98 (32.0%)	56 (25.7%)	
≥5/200 and <20/200	58 (18.9%)	59 (27.0%)	
<5/200	150 (49.0%)	93 (42.6%)	
Phthisis bulbi	32 (10.4%)	17 (7.8%)	0.036
Initial treatment^★^			<0.001
Number of articles reporting	5 (142 cases)	5 (171 cases)	
Palliative treatment^☆^	39 (27.5%)	0 (0%)	
Intravitreal antibiotics^#^	17 (12.0%)	65 (38.0%)	
Vitrectomy	86 (60.5%)	106 (61.9%)	

^★^Excluding those articles in which all cases of endophthalmitis were treated with PPV.

^☆^Treated with intravenous and topical antibiotics alone.

^#^Including those treated with vitrectomy after initial treatment with intravitreal antibiotics.

NLP: no light perception; LP: light perception; HM: hand movements; CF: counting fingers.

**Table 4:** Culture results of previous reports.

Author	Number of *Streptococcus* cases	Number of *Staphylococcus epidermidis* cases	Number of culture-positive eyes
Weinstein et al. [1]	2	0	7
Thordsen et al. [2]	2	2	4
Çakir et al. [3]	1	0	1
Al-Rashaed and Abu El-Asrar [4]	22	5	35
Alfaro et al. [5]	5	1	9

Total	32 (57.1%)	8 (14.3%)	56

Present study	10 (6.2%)	50 (31.2%)	160

**Table 5:** Previous reports of final visual acuity.

Author	NLP	LP-CF	≥5/200 and <20/200	≥20/200	Total
Thordsen et al. [2]	1	1	2	3	7
Çakir et al. [3]	0	3	0	1	4
Al-Rashaed and Abu El-Asrar [4]	11	10	0	10	31
Alfaro et al. [5]	2	2	1	7	12
Rabiah [39]	6	3	0	4	13
Jalali et al. [40]	8	2	0	7	17
Shilpa et al. [41]	4	0	3	3	10

Total	32 (34%)	21 (22.4%)	6 (6.4%)	35 (37.2%)	94

Present study	78 (11.7%)	255 (38.4%)	125 (18.9%)	206 (31.0%)	664

NLP: no light perception; LP: light perception; CF: counting fingers.
